# Hyaluronan synthesis by developing cortical neurons *in vitro*

**DOI:** 10.1038/srep44135

**Published:** 2017-03-13

**Authors:** Tania M. Fowke, Rashika N. Karunasinghe, Ji-Zhong Bai, Shawn Jordan, Alistair J. Gunn, Justin M. Dean

**Affiliations:** 1 Department of Physiology, Faculty of Medical and Health Sciences, University of Auckland, New Zealand

## Abstract

Hyaluronan is a linear glycosaminoglycan that forms the backbone of perineuronal nets around neurons in the cerebral cortex. However, it remains controversial whether neurons are capable of independent hyaluronan synthesis. Herein, we examined the expression of hyaluronan and hyaluronan synthases (HASs) throughout cortical neuron development *in vitro*. Enriched cultures of cortical neurons were established from E16 rats. Neurons were collected at days *in vitro* (DIV) 0 (4 h), 1, 3, 7, 14, and 21 for qPCR or immunocytochemistry. In the relative absence of glia, neurons exhibited HAS1–3 mRNA at all time-points. By immunocytochemistry, puncta of HAS2–3 protein and hyaluronan were located on neuronal cell bodies, neurites, and lamellipodia/growth cones from as early as 4 h in culture. As neurons matured, hyaluronan was also detected on dendrites, filopodia, and axons, and around synapses. Percentages of hyaluronan-positive neurons increased with culture time to ~93% by DIV21, while only half of neurons at DIV21 expressed the perineuronal net marker *Wisteria floribunda* agglutinin. These data clearly demonstrate that neurons *in vitro* can independently synthesise hyaluronan throughout all maturational stages, and that hyaluronan production is not limited to neurons expressing perineuronal nets. The specific structural localisation of hyaluronan suggests potential roles in neuronal development and function.

Hyaluronic acid (hyaluronan) is a linear glycosaminoglycan found in the extracellular matrix of most tissues throughout the body. Hyaluronan is produced by a family of three transmembrane hyaluronan synthases (HAS1–3)^1^,^2^,^3^, and exists most commonly as a high molecular weight molecule, with a size ranging from ≤3 × 10^6^ to ≥6 × 10^6^ Da^4^. Hyaluronan was originally described as an important structural scaffold for tissues, although there is increasing evidence for widespread roles in cellular signalling, differentiation, proliferation, and migration in peripheral organs and in the central nervous system (CNS)^5^,^6^.

Hyaluronan is widely expressed in the developing and adult CNS^6^,^7^,^8^,^9^,^10^. However, there are limited studies examining the cellular sources of hyaluronan in the brain^11^,^12^,^13^, and it is controversial whether neurons independently contribute to hyaluronan synthesis. Astrocytes are well established to produce hyaluronan, and express a range of HAS genes^11^,^12^,^14^,^15^. *In vitro*, astrocytes were also shown to form pericellular hyaluronan matrices, while cortical neurons in the same cultures did not^16^. More recently, however, hyaluronan was observed on a subset of mature parvalbumin-positive neurons in cortical cultures devoid of astrocytes^17^,^18^, suggesting potential for neuronal hyaluronan production.

In the cerebral cortex, as well as other grey matter regions, hyaluronan can form the backbone of specialised lattice-like extracellular matrix structures termed perineuronal nets, which surround the soma and proximal processes of mature GABAergic interneurons^19^,^20^,^21^,^22^,^23^,^24^. Perineuronal nets appear postnatally in the developing rodent and human brain^19^,^25^,^26^,^27^, and are associated with synaptic stability^28^,^29^ and closure of critical periods of plasticity^29^,^30^,^31^,^32^. However, the expression and function of hyaluronan on neurons without perineuronal nets is unclear.

In the present study, using enriched primary cultures of cortical neurons, we assessed the specific timing and structural localisation of hyaluronan and HASs throughout neuronal development.

## Methods

### Neuronal culture

Dissociated cultures of cortical neurons were generated from time-mated embryonic day 16 (E16) rat brains using established protocols^33^,^34^. The E16 rat brain is widely used for enriched cultures of cortical neurons, as the main period of gliogenesis in the rat cortex occurs after E17, and neuronal stem cells remain the predominant cell type until E19–20^35^. In brief, neurons were plated onto poly-D-lysine (80 μg/mL; Thermo Fisher Scientific, Auckland, NZ) and laminin (8 μg/mL; Sigma-Aldrich, Auckland, NZ) coated glass coverslips (Heinz Herenz Medizinalbedarf GmbH, Hamburg, Germany) in 24-well plates (1 × 10^5^ cells/well) for immunocytochemistry or 100 mm dishes (2 × 10^6 ^cells/dish) for mRNA analyses. Cultures were maintained in Neurobasal media containing B-27 Supplement (1X), Penicillin-Streptomycin (10 μg/mL), and GlutaMAX (1X) (Thermo Fisher Scientific). Neurons were collected at 4 h (day 0), and 1, 3, 7, 14, and 21 days *in vitro* (DIV) to cover key neuronal maturation stages, including dendrite, axon, and synapse development^36^,^37^,^38^. Neurons collected at DIV21 were treated with β-cytosine arabinofuranoside (AraC, 500 nM; Sigma-Aldrich) at DIV1 to inhibit glial proliferation associated with longer-term cultures^17^,^39^, and media was exchanged for non-AraC containing media at DIV3. The percentage of GFAP-positive astrocytes in the neuronal cultures was 0% at DIV0, DIV1, DIV3, and DIV7, <3% at DIV14, and <1% at DIV21. Using qPCR, only seven samples out of 23 showed detectable GFAP expression (at DIV0, DIV14, and DIV21), while when all time-points from DIV0–21 were combined, the average GFAP mRNA expression was >10,000-fold less than that for MAP2. All protocols were approved by the University of Auckland Animal Ethics Committee, and were performed in accordance with the New Zealand Government Animal Welfare Act.

### RNA isolation and quantitative real-time PCR

For each time point, three to four samples from independent cultures were obtained by pooling three 100-mm dishes per culture. Total RNA was extracted from cortical neurons using TRIzol according to the manufacturer’s protocol (Thermo Fisher Scientific), and samples were stored at −80 °C until use. RNA concentration and purity were assessed using the NanoDrop 2000 (Thermo Fisher Scientific).

For quantitative real-time PCR (qPCR), cDNA was synthesised from 1 μg total RNA using the iScript cDNA synthesis kit (Bio-Rad, Auckland, New Zealand) according to the manufacturer’s protocol. Experiments were performed using the QuantStudio 12 K Flex Real-Time PCR System (Thermo Fisher Scientific) with pre-optimised hydrolysis probe assays (Integrated DNA Technologies, Coralville, IA, USA; or Thermo Fisher Scientific). A minimum of three technical replicates were used per sample. Genes examined and details of probes used are shown in Table 1. All probes were exon-spanning and designed to detect no genomic DNA. GFAP and MAP2 probes were used to confirm culture purity (Table 2).

Data analysis was performed using QuantStudio software (Thermo Fisher Scientific), and calculations were performed in Microsoft Excel (Microsoft Co., Redmond, WA, USA). Relative gene expression was normalised to three endogenous controls, including β-actin, TATAA-box binding protein (TBP), and acidic ribosomal phosphoprotein P0 (ARBP) using global normalisation. mRNA expression was quantified by the ΔΔC_q_ (C_q_: quantification cycle) method^40^ using DIV0 neurons as reference samples. C_q_ values of the β-actin, TBP, and ARBP genes did not differ significantly between developmental time points or biological replicates (data not shown). These genes were previously reported as suitable qPCR reference genes for use in cortical neurons^41^.

### Immunocytochemistry

For optimal hyaluronan staining, neurons on coverslips were fixed in ethanol-acetic acid-formalin (70%, 5%, 4% v/v, respectively) in phosphate buffered saline (PBS) for 5 min at −20 °C^42^. Coverslips were then washed for 3 × 5 min in PBS, and blocked for 1 h in 5% bovine serum albumin (BSA)/PBS. Cells were incubated with biotinylated hyaluronic acid binding protein (bHABP, 1:500; #385911, Merck Millipore, NZ) and mouse monoclonal anti-microtubule-associated protein 2 (MAP2, 1:500; #M4403, Sigma-Aldrich) in PBS/3% BSA overnight at 4 °C. Cells were then washed for 3 × 5 min in PBS, and incubated in PBS/3% BSA with appropriate secondary antibodies (Thermo Fisher Scientific; 1:500 for each), including streptavidin-conjugated Alexa Fluor 594 and goat anti-mouse Alexa Fluor 488/660, for 2.5 h at room temperature, followed by Hoechst 33258 (1:10,000) to identify cell nuclei. Axons were also identified on neurons stained with bHABP/MAP2, and were classified as thin diameter, non-tapered processes that showed weak or no MAP2 immunoreactivity.

For HAS2–3 immunocytochemistry, neurons were fixed in 2% paraformaldehyde for 20 min. Cells were then washed 3 × 5 min in PBS, permeabilised in 0.1% Triton-X-100 in PBS for 10 min, washed twice in PBS, and then blocked in 5% NGS for 1 h. Neurons were incubated with rabbit polyclonal anti-HAS2 (1:100; #sc-66916, Santa Cruz Biotechnology) or rabbit polyclonal anti-HAS3 (1:200; #NBP1-86328, Novus Biologicals, Littleton, CO, USA), with anti-MAP2 (1:500; #M4403, Sigma-Aldrich) overnight at 4 °C. Phalloidin-488 (1:100; Thermo Fisher Scientific) was added with secondary antibodies for 2.5 h. The commercial HAS2 antibody is a rabbit polyclonal raised against amino acids 121–180 (DGN SED DLY MMD IFS EVM GRD KSA TYI WKN NFH EKG PGE TDE SHK ESS QHV TQL VLS NKS) within an internal region of HAS2 of human origin. The HAS3 antibody is a rabbit polyclonal raised against amino acids 127–186 (RQE DAY MLD IFH EVL GGT EQA GFF VWR SNF HEA GEG ETE ASL QEG MDR VRD VVR AST FSC) within an internal region of HAS3 of human origin. The full rat HAS2 (NCBI accession, AAB63209) and HAS3 (NCBI accession, NP_758822) amino acid sequences have 70% similarity, while the HAS2 and HAS3 sequences used to generate the antibodies have 40% similarity. Further, the HAS2 antibody amino acid sequence has only 42% similarity to rat HAS3, with no overlapping regions spanning more than four amino acids in length, while the HAS3 antibody amino acid sequence has only 41% similarity to rat HAS2, with no overlapping regions spanning more than four amino acids in length. The specificity of the HAS2 and HAS3 antibodies was further confirmed by western blot (see Supplementary Methods), which showed a single band at the expected molecular weight for each antibody (see Supplementary Fig. S1). Finally, to examine for potential cross-reactivity of the HAS2 and HAS3 antibodies, each antibody was pre-incubated with a blocking peptide (10:1 concentration, #NBP1-86328PEP; Novus Biologicals) against the HAS3 antibody; a blocking peptide for the HAS2 was not commercially available. By immunocytochemistry, there was a marked reduction in neuronal HAS3 staining when using the HAS3 antibody that was pre-incubated with the HAS3 blocking peptide, supporting the specificity of the HAS3 antibody (see Supplementary Fig. S2A,B). Further, a normal pattern of neuronal HAS2 staining was observed when using the HAS2 antibody that was pre-incubated with the HAS3 blocking peptide, suggesting no cross-reactivity of the HAS2 antibody with endogenous HAS3 protein (see Supplementary Fig. S2C,D).

For synaptic staining, cells were incubated with a combination of bHABP (1:500) and rabbit polyclonal anti-synaptophysin (pre-synaptic marker, 1:300; #ab68851, Abcam, Cambridge, MA, USA) or mouse monoclonal anti-PSD95 (post-synaptic marker, 1:200; #MA1-046, Affinity BioReagents, Golden, CO, USA).

Cells were washed for 3 × 5 min in PBS, and incubated in appropriate secondary antibodies, including streptavidin-conjugated Alexa Fluor 594 and goat anti-mouse Alexa Fluor 488/660, for 2.5 h at room temperature, followed by Hoechst 33258 to identify cell nuclei. In preliminary experiments using goat polyclonal anti-HAS1 antibody (1:100; #sc-23145, Santa Cruz Biotechnology, Dallas, TX, USA), we were unable to produce optimal HAS1 staining. To detect perineuronal nets, neurons were stained with the established chondroitin sulphate proteoglycan marker biotinylated *Wisteria floribunda* agglutinin (WFA, 1:500; #L1516, Sigma-Aldrich)^19^ and MAP2 using the procedures described above. Omission of primary antibodies/binding proteins resulted in no positive staining.

After 3 × 5 min washing in PBS, coverslips were mounted onto glass slides using Prolong Gold Antifade medium (Thermo Fisher Scientific), cured overnight at room temperature, and then sealed. The specificity of hyaluronan staining was confirmed by pre-treatment of fixed neurons with hyaluronidase from *Streptomyces hyalurolyticus* (4 U/mL, 37 °C for 1 h; Sigma-Aldrich), which eliminated the HABP signal (see Supplementary Fig. S3)^43^. Neurons were imaged using an Olympus FV1000 confocal microscope (Olympus Co., Center Valley, PA, USA) under a 60X (N.A. 1.35) or 100X (N.A. 1.4) objective, while a Zeiss AxioImager M2 fluorescent microscope (Carl Zeiss, Thornwood, NY, USA) under a 20X (N.A. 0.5) objective was used for cell counts. Combined differential interference contrast (DIC) and fluorescent images were also obtained using the Olympus FV1000 confocal microscope.

### Quantitative analysis of hyaluronan expression

Assessment of the percentage of cortical neurons that expressed hyaluronan was performed on neurons double-labelled with MAP2 and bHABP. A circular region of interest was traced 1000 μm inside the outer edge of each coverslip, and 10 random sites were imaged (Zeiss AxioImager M2). MAP2-positive neurons were then classified as either positive or negative for hyaluronan, and counts were obtained for each site using ImageJ^44^.

### Statistical analysis

For qPCR experiments, data are presented as relative expression values (RQ) compared to DIV0 of culture. One-way analysis of variance (ANOVA) followed by Dunnett’s multiple comparisons test was used to assess differences between the reference group (DIV0) and other time points using ΔC_q_ values. One-way ANOVA with Tukey’s multiple comparisons post-hoc test was used to analyse differences in the percentages of hyaluronan-positive neurons between time points in culture. A value of *P* < 0.05 was considered statistically significant. Graphs were generated using Prism software (GraphPad Software, Inc., La Jolla, CA, USA).

## Results

### Cultured cortical neurons express the family of hyaluronan synthases throughout development *in vitro*

#### mRNA expression by qPCR

Cultured cortical neurons expressed HAS1, HAS2, and HAS3 mRNA at all time points. Using normalised ΔC_q_ data, HAS3 was expressed at the highest levels (~670 fold higher than HAS1), followed by HAS2 (~130 fold greater than HAS1), while HAS1 consistently had the lowest expression (Table 2).

**Figure 1:**
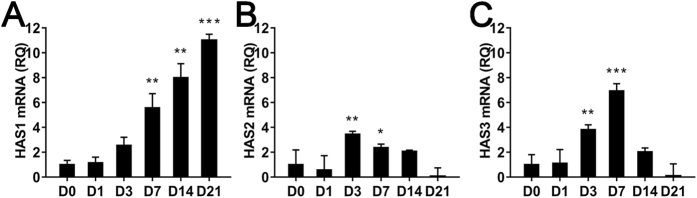
Hyaluronan synthase (HAS)1–3 mRNA expression during cortical neuron development *in vitro*. Gene expression was normalised to days *in vitro* (DIV)0 to provide relative quantification values (RQ). *N = *3–5 samples from 3–4 independent cultures for each developmental stage. Error bars are minimum and maximum RQ; *P < 0.05, **P < 0.01, ***P < 0.001 relative to DIV0.

There was a significant overall effect of time in culture on HAS1–3 gene expression (one-way ANOVA, *P  *< 0.03). Thus, we assessed the fold-change in target gene expression over time in culture. Relative to DIV0, HAS1 and HAS3 mRNA expression progressively increased to peak at DIV21 and DIV7, respectively (Fig. 1A,C), while HAS3 expression progressively decreased thereafter. HAS2 expression peaked at DIV3, and decreased thereafter (Fig. 1B).

#### Protein expression by immunocytochemistry

**Figure 2:**
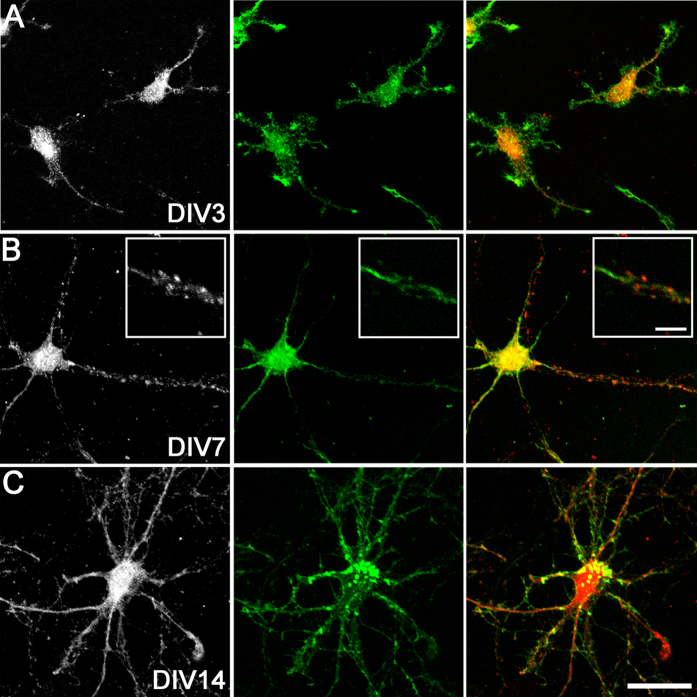
HAS2 protein expression on cortical neurons *in vitro*. HAS2 (left: grey, right: red) and actin (green) at DIV3 (**A**), DIV7 (**B**), and DIV14 (**C**). A high magnification example of a neuronal process expressing HAS2 is shown (**B**, inset). Scale bar: 5 μm (inset), 30 μm.

**Figure 3:**
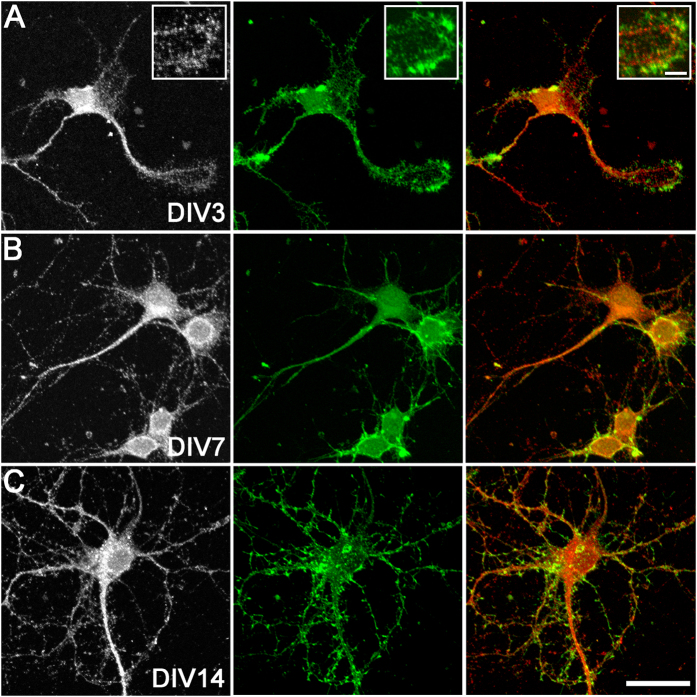
HAS3 protein expression on cortical neurons *in vitro*. HAS3 (left: grey, right: red) and actin (green) at DIV3 (**A**), DIV7 (**B**), and DIV14 (**C**). A high magnification example of a growth cone expressing HAS3 is shown (**A**, inset). Scale bar: 5 μm (inset), 30 μm.

Next, we assessed the expression of HAS2 and HAS3 proteins on MAP2-positive cortical neurons by immunocytochemistry. At DIV0, immature neurons showed limited somatic HAS2 and HAS3 immunoreactivity, while by DIV3, most neurons exhibited a punctate pattern of HAS2 (Fig. 2) and HAS3 (Fig. 3) on the cell soma and processes. With neuronal maturation (DIV7 and DIV14), more extensive HAS2 and HAS3 protein expression was observed on neuronal processes (e.g., Fig. 2B). At early developmental stages (up until DIV7), there was also evidence of HAS2 and HAS3 expression on actin-positive lamellipodia, growth cones, and filopodia (e.g., Fig. 3A).

### Cortical neurons produce hyaluronan throughout development *in vitro*

To determine whether the neuronal HASs were functional, we assessed the expression of hyaluronan on cortical neurons using biotinylated hyaluronic acid binding protein (bHABP), which selectively labels hyaluronan ≥10 monosaccharides in size^45^. The percentages of hyaluronan-positive neurons over time in culture are shown in Table 3.

**Figure 4:**
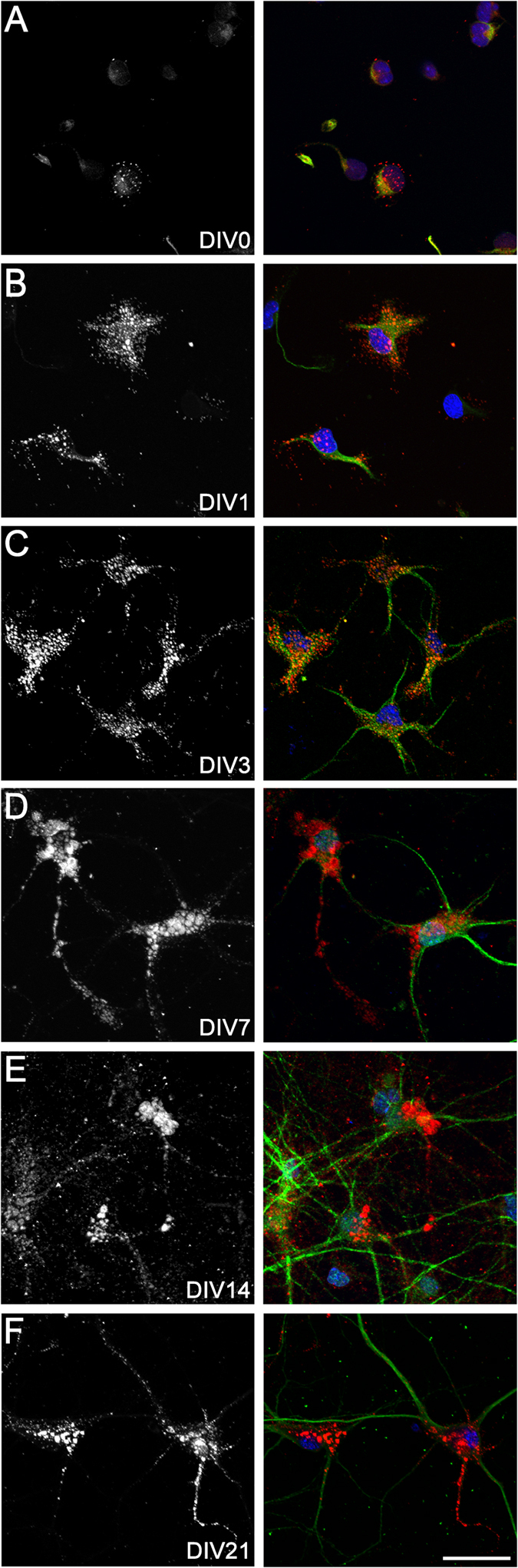
Hyaluronan expression on cortical neurons *in vitro*. Neurons at DIV0, 1, 3, 7, 14, and 21 (**A**–**F**) were stained with biotinylated hyaluronic acid binding protein (bHABP) (left: grey, right: red), MAP2 (green), and Hoechst 33258 (blue). Scale bar: 30 μm.

At 4 h after culture, ~16% of cortical neurons showed a pattern of small hyaluronan puncta on cell bodies (Fig. 4A). At DIV1 and DIV3, neurons showed a more extensive pattern of pericellular hyaluronan surrounding the cell body and extending along the length of developing neurites (Fig. 4B,C). By DIV7, DIV14, and DIV21, almost all neurons expressed hyaluronan (Table 3), with larger puncta localised to the cell bodies, and smaller puncta along processes (Fig. 4D,E). Additionally, from DIV14, more diffuse hyaluronan was present in the pericellular and extracellular regions. Pre-treatment of cultured neurons with hyaluronidase from *Streptomyces hyalurolyticus* abolished hyaluronan staining (see Supplementary Fig. S3).

### Hyaluronan is localised to specific neuronal structures *in vitro*

**Figure 5:**
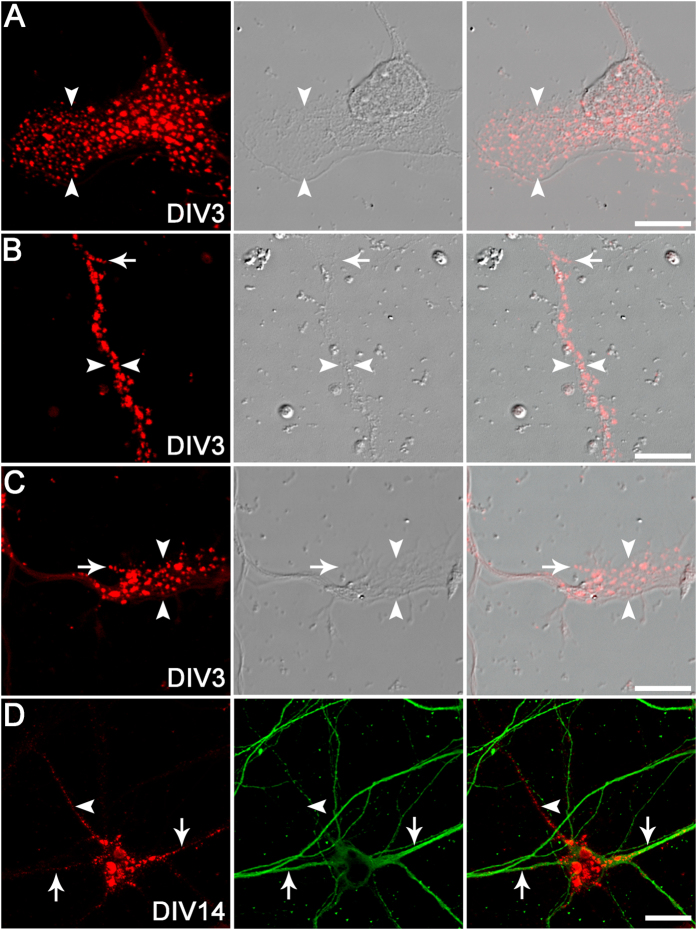
Structural localisation of hyaluronan on cortical neurons *in vitro*. Subcellular structures were visualised under differential interference contrast (DIC) optics. bHABP localisation (red) was observed on neuronal lamellipodia (arrowheads; **A**), immature neurites (between arrowheads; **B**), growth cones (arrowheads; **C**), filopodia (arrows; **B**,**C**), dendrites (arrows; **D**), and axons (defined as a thin, MAP2-negative process, arrowheads; **D**). MAP2, green. Scale bars: 10 μm (**D**), 30 μm (**A**–**C**).

**Figure 6:**
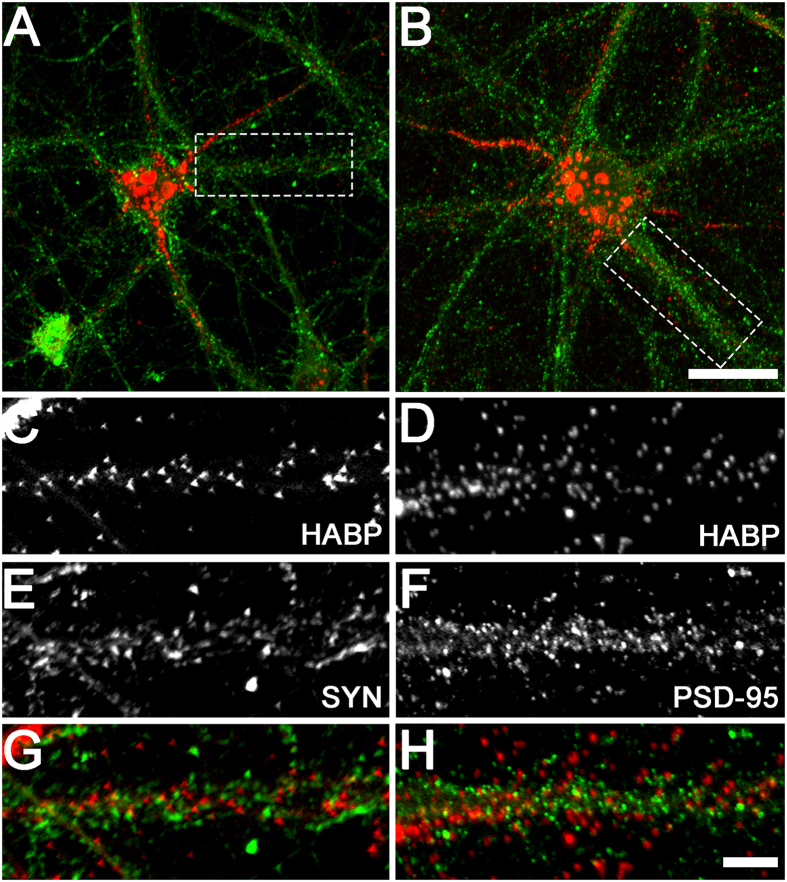
Spatial relationship between hyaluronan and synaptic markers on cortical neurons *in vitro*. Representative examples of neurons labelled with hyaluronan (red) and synaptophysin (**A**, green) or PSD-95 (**B**, green) at DIV21 are shown. High magnification images indicate small spatial separation, but no colocalisation, between hyaluronan (**C**,**D**) and synaptophysin (SYN; **E**,**G**) or PSD-95 (**F**,**H**) immunoreactive puncta on neuronal processes. Scale bars: 5 μm (**A**,**B**), 20 μm (**C**–**H**).

To confirm our findings of HAS expression on specific neuronal structures, we examined the detailed expression patterns of neuronal hyaluronan. Hyaluronan was frequently localised to neuronal lamellipodia (Fig. 5A), immature neurites (Fig. 5B,C), and growth cones (Fig. 5C) in developing neurons from DIV1–7 (only DIV3 images are shown), with occasional expression on filopodia (Fig. 5B,C). In more mature neurons, hyaluronan was also localised to dendrites and axons (Fig. 5D). In addition, we examined the spatial association of neuronal hyaluronan with the mature synapse by comparing hyaluronan labelling with immunoreactivity for presynaptic (synaptophysin) and postsynaptic (PSD-95) markers on mature neurons. Both presynaptic boutons and postsynaptic sites were closely associated with hyaluronan matrix along neuronal processes, although we did not observe any direct overlap of hyaluronan with synaptic markers (Fig. 6).

### Development of mature perineuronal net components on cortical neurons *in vitro*

**Figure 7:**
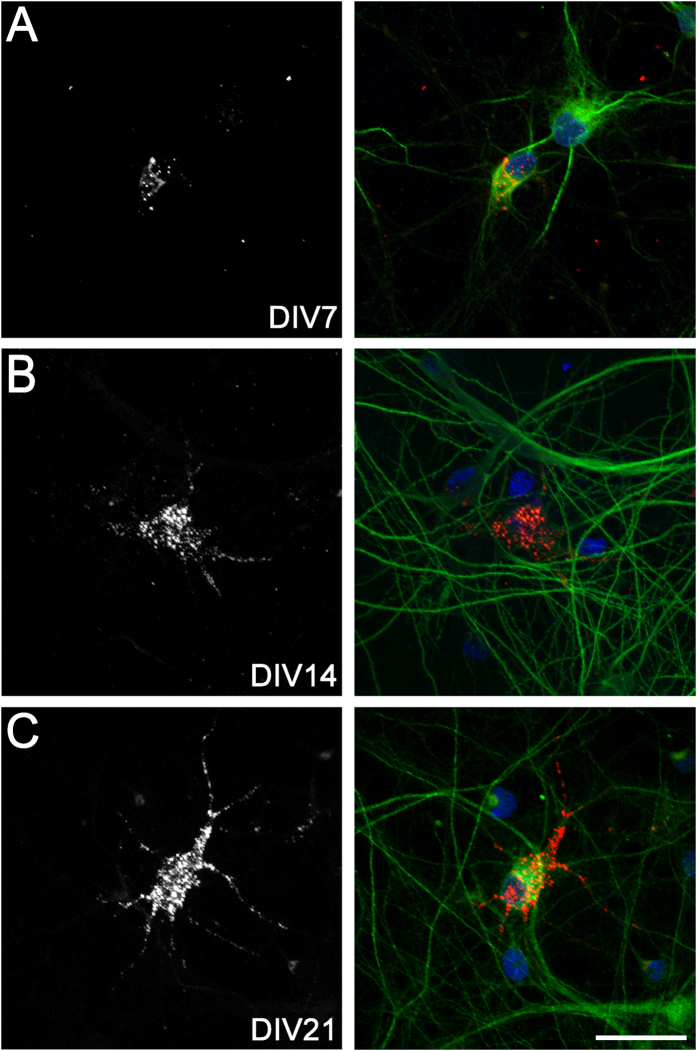
Perineuronal net expression on cortical neurons *in vitro*. Neurons at DIV7, 14, and 21 (**A**–**C**) were stained with biotinylated WFA (left: grey, right: red), MAP2 (green), and Hoechst 33258 (blue). Scale bar: 30 μm.

Finally, we assessed the timing of formation of perineuronal net-like structures in our culture system using WFA, which labels the N-acetylgalactosamine component of chondroitin sulphate chains and is commonly used to identify mature perineuronal nets^19^. The percentages of WFA*-*positive neurons over time in culture are shown in Table 3. There was no evidence of WFA expression on cortical neurons at DIV0–3 (Table 3). However, at DIV7, DIV14, and DIV21, ~7%, ~17%, and 53%, respectively, of all cortical neurons expressed pericellular WFA. This contrasts with our finding that almost all neurons (>90%) showed hyaluronan labelling from DIV7–DIV21. At DIV7, only a few small WFA-positive puncta were visible on neuronal cell bodies, with no obvious process staining (Fig. 7A). By DIV14 there were larger WFA-positive puncta localised to neuronal cell bodies, and expression of smaller puncta along processes (Fig. 7B). By DIV21, there was a more extensive pattern of pericellular WFA (Fig. 7C), in a pattern closely resembling a classical mesh-like perineuronal net structure^18^,^22^.

## Discussion

There is now strong evidence that perineuronal nets can control CNS plasticity, particularly in the cerebral cortex and hippocampus^29^. Hyaluronan is a major component of the extracellular matrix in the brain, and contributes to perineuronal net-mediated regulation of neuronal signalling and synaptic plasticity in developmentally mature neurons^46^,^47^,^48^. Herein, we provide new *in vitro* evidence that cortical neurons express the entire family of HAS enzymes, and produce hyaluronan on multiple structures important for neuronal development and synaptic function.

Astrocytes are a major source of hyaluronan in the brain^14^,^15^, and contribute to the formation of perineuronal nets *in vitro* and *in vivo*^13^,^24^. Indeed, in co-cultures with cortical neurons, astrocytes produced a pericellular hyaluronan matrix by DIV7, while the neurons did not^16^. However, in longer recovery experiments in purified neuronal cultures, a small subset of cortical neurons expressed pericellular hyaluronan in a classical perineuronal net-like pattern at DIV12 and DIV21^17^,^18^. Further, cultured hippocampal neurons expressed hyaluronan at DIV24, but not at DIV10^46^. By contrast, in our enriched neuronal cultures largely devoid of astrocytes, we found punctate hyaluronan expression on ~16% of immature cortical neurons as early as 4 h *in vitro*, while ~85% of neurons expressed hyaluronan by DIV3, and >90% at DIV7–21. Further, only ~17% of DIV14 neurons and 53% of DIV21 neurons were positive for WFA, a marker of perineuronal net-like structures present on mature cortical neurons^19^. Overall, these findings suggest that hyaluronan expression is not limited to neurons with perineuronal nets, and that hyaluronan expression appears much earlier in the maturation of post-mitotic neurons than previously reported.

We observed abundant hyaluronan puncta on multiple structures on immature neurons, including cell bodies and developing neurites. The function of this hyaluronan is unclear. However, growth of dorsal root ganglion neurons on a hyaluronan substrate reduces neurite extension^49^. Further, the major hyaluronan receptor CD44 is expressed on neuronal dendrites, and blockade of CD44 increases dendritic arborisation of cortical and hippocampal neurons both *in vitro* and *in vivo*^50^. Conversely, blockade of RHAMM, another hyaluronan receptor, inhibits neurite extension in cultures of primary neurons and other neural cell lines^51^. Thus, hyaluronan located on developing neurites may be important for neurite outgrowth, potentially through receptor-mediated signalling pathways. Indeed, hyaluronan signalling through the CD44 receptor regulates various cytoskeleton-mediated processes, including motility in astrocytes and adhesion and metastasis in cancer cells^52^,^53^.

We also found evidence of hyaluronan expression on lamellipodia and filopodia of immature cortical neurons *in vitro*. Although there are no comparable studies in the brain, hyaluronan is found on lamellipodia and filopodia of oesophageal carcinoma cells and epidermal keratinocytes, where it plays a key role in outgrowth and adhesion^54^,^55^. Further, addition of hyaluronan induces lamellipodia outgrowth in mouse mammary epithelial cells^56^. Interestingly, growth of chick dorsal root ganglion neurons on a substrate of synthetic hyaluronan reduces growth cone extension^49^. In addition, CD44 expression enhances filopodia growth of neuroblastoma cells^57^. Thus, hyaluronan may play an important role in controlling cytoskeletal adhesion and outgrowth in early stage cortical neurons.

Hyaluronan was also located along axons of more mature neurons (i.e., from DIV7–DIV21 of development). Although axonal localisation of hyaluronan in cortical neurons has not been reported, hyaluronan is expressed in axons of granule cells in the rat cerebellum^45^ and surrounds axons in the white matter and spinal cord^8^. Other perineuronal net components such as brevican are expressed at particularly high levels on the axon initial segment of mature hippocampal neurons^58^ and motor neurons in the superior colliculus^59^. ‘Axonal coats’ of aggrecan are also present in various human basal ganglia regions^60^, in the human lateral geniculate body^61^, and in the rat thalamus^62^, often independently of perineuronal nets. Interestingly, there is evidence that hyaluronan can control axonal outgrowth of optic tract and locus ceruleus neurons^63^,^64^,^65^. Further, removal of hyaluronan promotes regeneration of severed axons in the rat brain^66^, although addition of low molecular weight hyaluronan oligosaccharides enhances axonal regrowth following spinal cord injury^67^. Overall, these data suggest potential roles of hyaluronan in axon development and function.

In mature cortical neurons (DIV21), we observed a close spatial association of hyaluronan with synaptic contact sites. In mature hippocampal neuronal cultures, hyaluronan enwraps spine necks^46^, while synapses are also embedded in brevican and neurocan, both chondroitin sulphate proteoglycan components of mature perineuronal nets^58^. In the present study, we found no obvious overlap of hyaluronan with synaptophysin or PSD-95, which is supported by evidence that perineuronal net components enclose synapses, but are absent from the sites of synaptic contact^46^,^58^. This synaptic association of hyaluronan on cortical neurons supports its role as part of the perisynaptic matrix, which may stabilise synaptic architecture and assist in maintaining the synaptic microenvironment by regulating receptor trafficking^46^, ion diffusion^68^, and neurotransmitter spillover^69^. Indeed, removal of hyaluronan using hyaluronidase impairs voltage-dependent calcium currents and long-term potentiation in brain slices^48^, and fear memory *in vivo*^48^,^70^. Similarly, disruption of the chondroitin sulphate proteoglycan components of perineuronal nets using chondroitinase impairs fear memory^71^, synaptic stability, and postsynaptic currents^72^, prevents critical period closure^31^, and enhances recognition memory and long-term depression^73^. Together, these studies implicate hyaluronan, both alone and as a major perineuronal net scaffold, in the control of synaptic plasticity.

The capacity of cultured cortical neurons to independently produce hyaluronan was confirmed by robust expression of HAS2 and HAS3 mRNA. Further, HAS2 and HAS3 protein expression coincided with the widespread distribution of hyaluronan throughout various neuronal structures. By contrast, expression of HAS1 mRNA was very low, and we were unable to detect HAS1 protein by immunocytochemistry. Although there are limited studies of HAS expression in the CNS, HAS2 and HAS3 mRNA (but not HAS1) are expressed in the rat embryonic brain and in selected neurons in the rat visual cortex and cerebellum^13^,^74^, while HAS1 and HAS3 mRNA are found in developing spinal cord neurons^75^, and HAS3 mRNA in cultured cortical neurons at DIV8^17^. HAS1 and HAS2 proteins are also expressed in neurons following grey matter stroke in humans^76^. The specific contributions of HAS1–3 to neuronal hyaluronan production are unknown. However, in cultured HEK cells, HAS2 or HAS3 expression results in a larger pericellular hyaluronan matrix compared with HAS1^77^,^78^. Interestingly, HAS1 and HAS2 synthesise higher molecular weight hyaluronan (2 × 10^5^–2 × 10^6^ Da) compared with HAS3 (1 × 10^5^ Da–1 × 10^6^ Da)^79^. Further, the biological effects of hyaluronan are dependent on its size, through activation of different receptor-mediated signalling pathways^80^,^81^. Thus, differing patterns of HAS expression and molecular weights of hyaluronan on neurons may serve a range of biological functions depending on the timing of their expression.

The delayed (DIV7) but progressive increase in punctate WFA labelling observed on neuronal cell bodies and processes in the present study is consistent with that previously reported *in vitro*^18^, and with the delayed formation of perineuronal nets in the rat cerebral cortex *in vivo*^25^; in that study, weak perineuronal nets were first observed at postnatal day 14, and then progressively increased until postnatal day 40^25^. WFA labels the N-acetylgalactosamine component of chondroitin sulphate chains on mature perineuronal nets^19^, including neurocan, versican, brevican, aggrecan, and phosphacan. These components are linked to the hyaluronan backbone via link proteins to form the lattice-like perineuronal net structure. Although we did not examine the individual expression of these perineuronal net components in the present study, their specific development on neurons has been previously characterised *in vitro* and *in vivo*^17^,^18^,^26^,^74^,^82^,^83^, with evidence for both neuronal- and glial-dependent synthesis^13^,^17^,^18^,^58^,^75^.

In conclusion, these data suggest that cortical neurons have the capacity to independently synthesise hyaluronan on multiple neuronal structures throughout their development. The specific cellular functions of this neuronal hyaluronan may depend on the stage of neuronal maturation, as well as the co-expression of perineuronal net components. Further studies are required to determine the specific roles of hyaluronan in neuronal development and function.

## Additional Information

**How to cite this article**: Fowke, T. M. *et al*. Hyaluronan synthesis by developing cortical neurons *in vitro. Sci. Rep.* **7**, 44135; doi: 10.1038/srep44135 (2017).

**Publisher's note:** Springer Nature remains neutral with regard to jurisdictional claims in published maps and institutional affiliations.

## Supplementary Material

Supplementary Information

## Figures and Tables

**Table 1:** Hydrolysis probes used for gene expression analysis.

Gene	Assay code
ARBP (NM_022402)	Rn.PT.58.45174577Probe: 5′-/56-FAM/CGTGATGCC/ZEN/CAGGGAAGACAGG/3IABkFQ/-3′Primer 1: 5′-GAAGCATTTTGGGTAGTCATCC-3′Primer 2: 5′-GTCCTCATTAGAGTGACATCGTC-3′
β-actin (NM_031144)	Rn.PT.58.10607869Probe: 5′-/56-FAM/CCGCCACCA/ZEN/GTTCGCCATG/3IABkFQ/-3′Primer 1: 5′-GGAGCCGTTGTCGACGA-3′Primer 2: 5′-AGTACAACCTTCTTGCAGCTC-3′
GFAP (NM_017009)	Rn.PT.58.36145160Probe: 5′-/56-FAM/CAACCTCCA/ZEN/GATCCGAGAAACCAGC/3IABkFQ/-3′Primer 1: 5′-CATCTCCACCGTCTTTACCAC-3′Primer 2: 5′-AACCGCATCACCATTCCTG-3′
HAS1 (NM_172323)	Rn.PT.58.11307716Probe: 5′-/56-FAM/AGTCACAGA/ZEN/CCTGCACGTAGTCCA/3IABkFQ/-3′Primer 1: 5′-CTTCATCCAGCACTCGCA-3′Primer 2: 5′-TCATGTACACGGCTTTCAAGG-3′
HAS2 (NM_013153)	Rn.PT.58.33810760Probe: 5′-/56-FAM/ACATAATCC/ZEN/ACGCTTCTGCCCAGT/3IABkFQ/-3′Primer 1: 5′-GACCTTCACCATCTCCACAF-3′Primer 2: 5′-AAGTCATGTACACCGCCTTC-3′
HAS3 (NM_172319)	Rn.PT.58.10107850Probe: 5′-/56-FAM/ACCCAGCCT/ZEN/GCACCATTGAGA/3IABkFQ/-3′Primer 1: 5′-CTCCTCCAACACCTCCTACT-3′Primer 2: 5′-GGCAACTCAGTGGACTACATC-3′
MAP2 (NM_013066)	Rn01401429_m1 (Thermo Fisher Scientific)Probe context sequence: GG-ACCACCAGGT-CAGAACCAAT-TCG
TBP (NM_001004198)	Rn.PT.58.18641244Probe: 5′-/56-FAM/ACTCCTGCC/ZEN/ACACCAGCCTC/3IABkFQ/-3′Primer 1: 5′-CAAGTTTACAGCCAAGATTCACG-3′Primer 2: 5′-TTCACCAATGACTCCTATGACC-3′

ARBP, Acidic ribosomal phosphoprotein P0; GFAP, glial fibrillary acidic protein; HAS1–3, hyaluronan synthase 1–3; MAP2, microtubule-associated protein 2; TBP, TATAA-box binding protein. All probes were sourced from Integrated DNA Technologies unless otherwise stated. Note that only the context sequence for the MAP2 probe was provided by ThermoFisher Scientific.

**Table 2:** Average ΔC_q_ values of HAS1–3, MAP2, and GFAP over time in culture.

	HAS1	HAS2	HAS3	MAP2	GFAP
DIV0	17.57 ± 0.22	10.84 ± 0.59	8.32 ± 0.40	5.35 ± 0.64	18.88
DIV1	17.36 ± 0.26	11.52 ± 0.66	8.21 ± 0.64	5.83 ± 0.74	n.d.
DIV3	16.27 ± 0.32	8.69 ± 0.12	6.23 ± 0.19	3.58 ± 0.17	n.d.
DIV7	15.41 ± 0.57	9.24 ± 0.14	5.43 ± 0.29	3.31 ± 0.40	n.d.
DIV14	14.77 ± 0.64	9.42 ± 0.05	7.13 ± 0.30	2.59 ± 0.18	16.85
^a^DIV21	14.13 ± 0.29	14.78 ± 0.40	11.99 ± 0.57	5.37 ± 0.50	13.99 ± 0.45

Data are mean ± SEM. *N = *3–5 samples per developmental stage (from 3–4 independent cultures). ΔC_q_ values reflect quantitative real-time PCR (qPCR) amplification cycle differences between the target genes and endogenous controls. Lower ΔC_q_ values reflect higher gene expression. DIV, days *in vitro;* n.d., not detected. Note that GFAP was only expressed in 2/5 samples at DIV0 and 2/4 at DIV14. ^a^DIV21 neurons were treated with β-cytosine arabinofuranoside (AraC) to inhibit glial proliferation.

**Table 3:** Percentage of neurons expressing hyaluronan or *Wisteria floribunda* agglutinin throughout development *in vitro*.

	DIV0	DIV1	DIV3	DIV7	DIV14	DIV21
%HA^+^	16.2 ± 1.3	60.4 ± 1.8^†^	84.2 ± 1.3^†^	91.5 ± 1.0^†^	97.4 ± 0.5*	92.6 ± 2.5
%WFA^+^	—	—	—	7.5 ± 1.3	16.8 ± 2.3	53.4 ± 4.3^†^

Neuronal hyaluronan (HA) and *Wisteria floribunda* agglutinin (WFA) expression were confirmed by MAP2 co-labelling. Data are mean ± SEM, *P < 0.05, ^†^*P *< 0.001, *vs*. previous culture time point. *N *= > 20 sites counted per time point from 2–3 independent cultures.
