# Supplementary material for: Hyaluronan synthesis by developing cortical neurons in vitro
Source: Sci Rep. 2017 Mar 13;7:44135. doi: 10.1038/srep44135 (PMC5347017; doi:10.1038/srep44135)
Supplement: Supplementary Information [file srep44135-s1.pdf]

Hyaluronan synthesis by developing cortical neurons *in vitro*

Tania M. Fowke[1], Rashika N. Karunasinghe[1], Ji-Zhong Bai[1], Shawn Jordan[1], Alistair J. Gunn[1], Justin M. Dean*[1]

[1]Department of Physiology, Faculty of Medical and Health Sciences, University of Auckland, New Zealand

***Corresponding author:** Justin M. Dean, Ph.D.

Department of Physiology, Faculty of Medical and Health Sciences, 85 Park Road, Grafton, Auckland 1010, New Zealand

Phone: +64 9 373 7599 ext 86201; Email: j.dean@auckland.ac.nz

Supplementary Information

Supplementary Methods

Western blot for HAS2 and HAS3 antibody validation

Adult rat testes tissue was homogenised with a rotor homogeniser in radioimmunoprecipitation assay buffer (RIPA) (50 mM Tris HCl pH 8.0, 150 mM NaCl, 0.1% Triton-X-100, 0.5% sodium deoxycholate, 0.1% sodium dodecyl sulphate), with a cocktail of protease and phosphatase inhibitors (Roche, Basel, Switzerland), and incubated at 4ºC with constant agitation for 2 h. The lysate was centrifuged for 20 min at 14,000 x *g*, and the supernatant was collected. Protein concentration was determined using the Bio-Rad DC protein assay (Bio-Rad). Protein was incubated in Laemmli buffer (Bio-Rad) for 5 min at 95ºC. For each sample, 30 µg of protein was loaded onto AnykD Mini-Protean TGX Stain-Free gels (Bio-Rad), with a Precision Plus WesternC Protein Standard (Bio-Rad). The gel was run at 50 V for 5 min, followed by 120 V for 80 min, at room temperature. Proteins were transferred onto a nitrocellulose membrane using the Transblot Turbo system (mixed molecular weight program, Bio-Rad). Membranes were blocked in 5% skim milk/tris-buffered saline, 0.1% Tween (TBST) for 1 h, and then incubated in rabbit anti-HAS2 (1:100, Santa-Cruz Biotechnology) or rabbit anti-HAS3 (1:200, Novus Biologicals) in 3% skim milk/TBST overnight at 4ºC. Membranes were washed for 3 x 10 min in TBST, incubated in biotinylated goat anti-rabbit antibody (1:5000, Vector Laboratories, Burlingame, CA, USA) for 1 h at room temperature, washed for 3 x 10 min in TBST, and then incubated with Streptactin-HRP (1:20,000, Bio-Rad) for 1 h at room temperature. Finally, membranes were washed for 3 x 10 min, and then

incubated in Clarity Western ECL Substrate (Bio-Rad) for 5 min. Protein bands were visualised using the ChemiDoc system (Bio-Rad).

Supplementary Figures

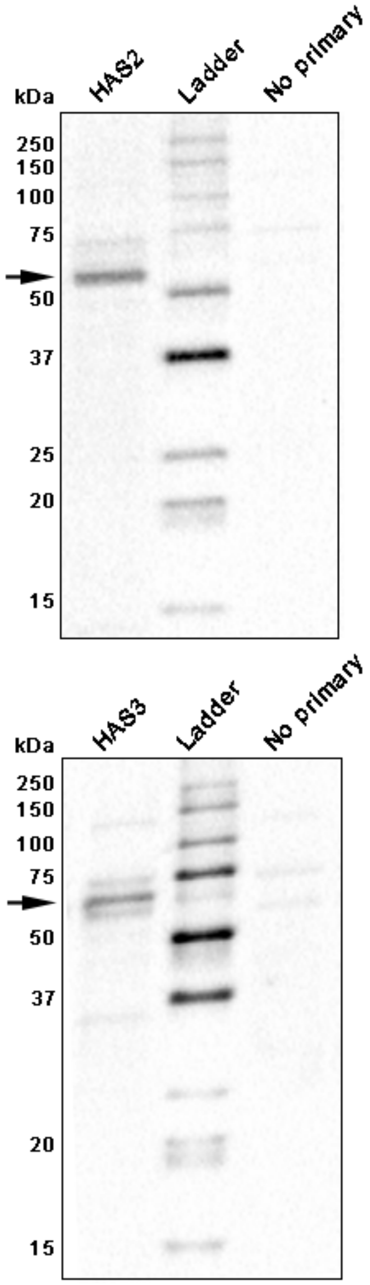

Supplementary Figure S1. Western blot showing HAS2 (top, lane 1) and HAS3 (bottom, lane 1) protein expression in testes lysate. Note the appearance of a single dark band for HAS2 and HAS3 (arrows, approximately 63 kDa). There was minimal background staining produced by incubation with secondary and tertiary antibodies (lane 3). A 10–250 kDa ladder is shown in lane 2. The exposure time was 2 s for both membranes.

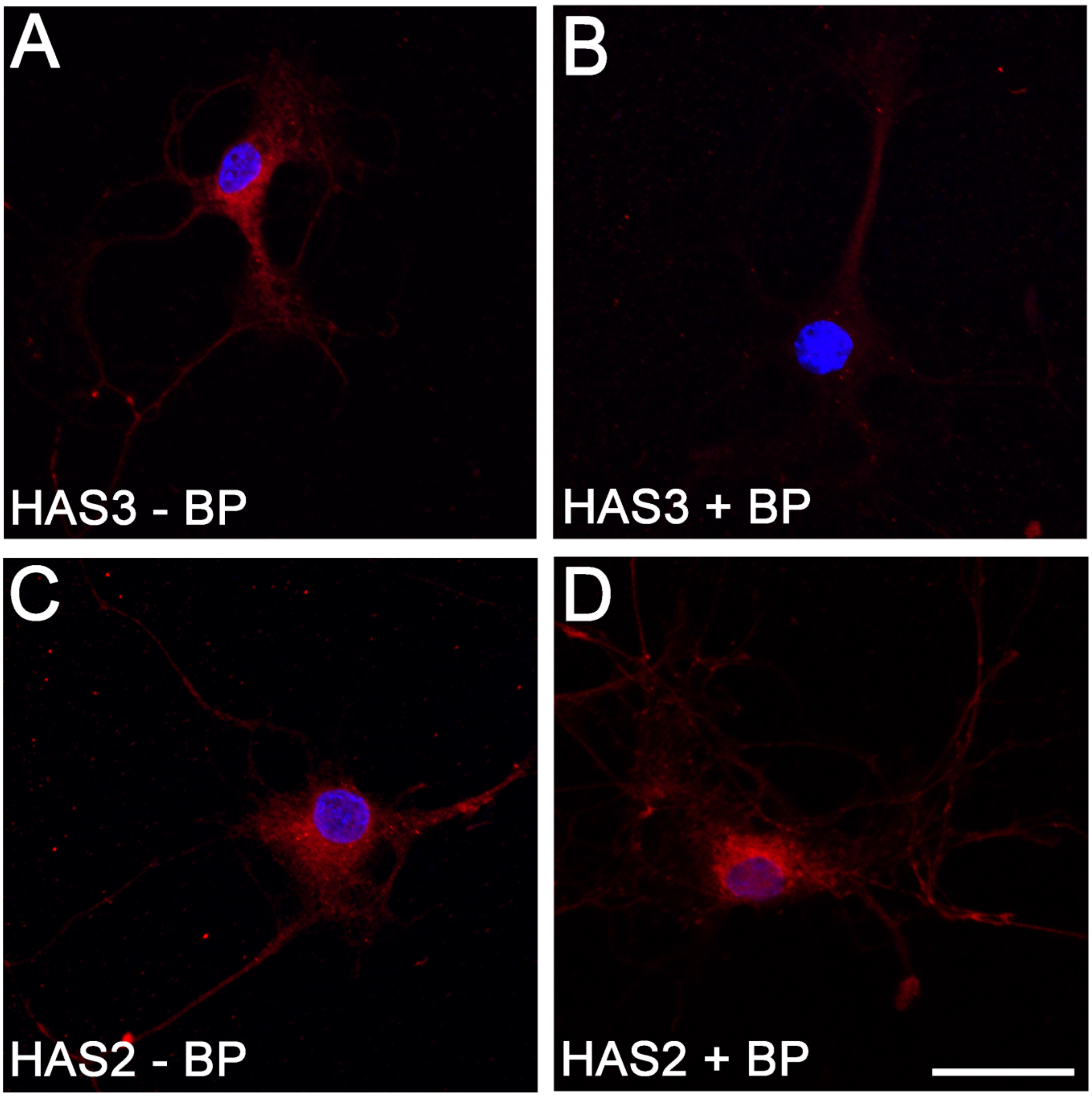

Supplementary Figure S2. HAS2 and HAS3 labelling of cortical neurons after pre-incubation of antibodies with a HAS3 blocking peptide. Representative examples from neurons at DIV7 are shown. (A) HAS3 labelling without blocking peptide pre-incubation. (B) HAS3 staining after pre-incubation of the HAS3 antibody with the HAS3 blocking peptide. Note the reduction in red HAS3 immunoreactivity on the neuronal cell body and processes after antibody blocking. (C) HAS2 labelling without blocking peptide pre-incubation. (D) HAS2 staining after pre-incubation of the HAS2 antibody with the HAS3 blocking peptide. Note that HAS2 labelling was not changed with HAS3 blocking peptide pre-incubation. Scale bar: 30 μm; BP, blocking peptide.

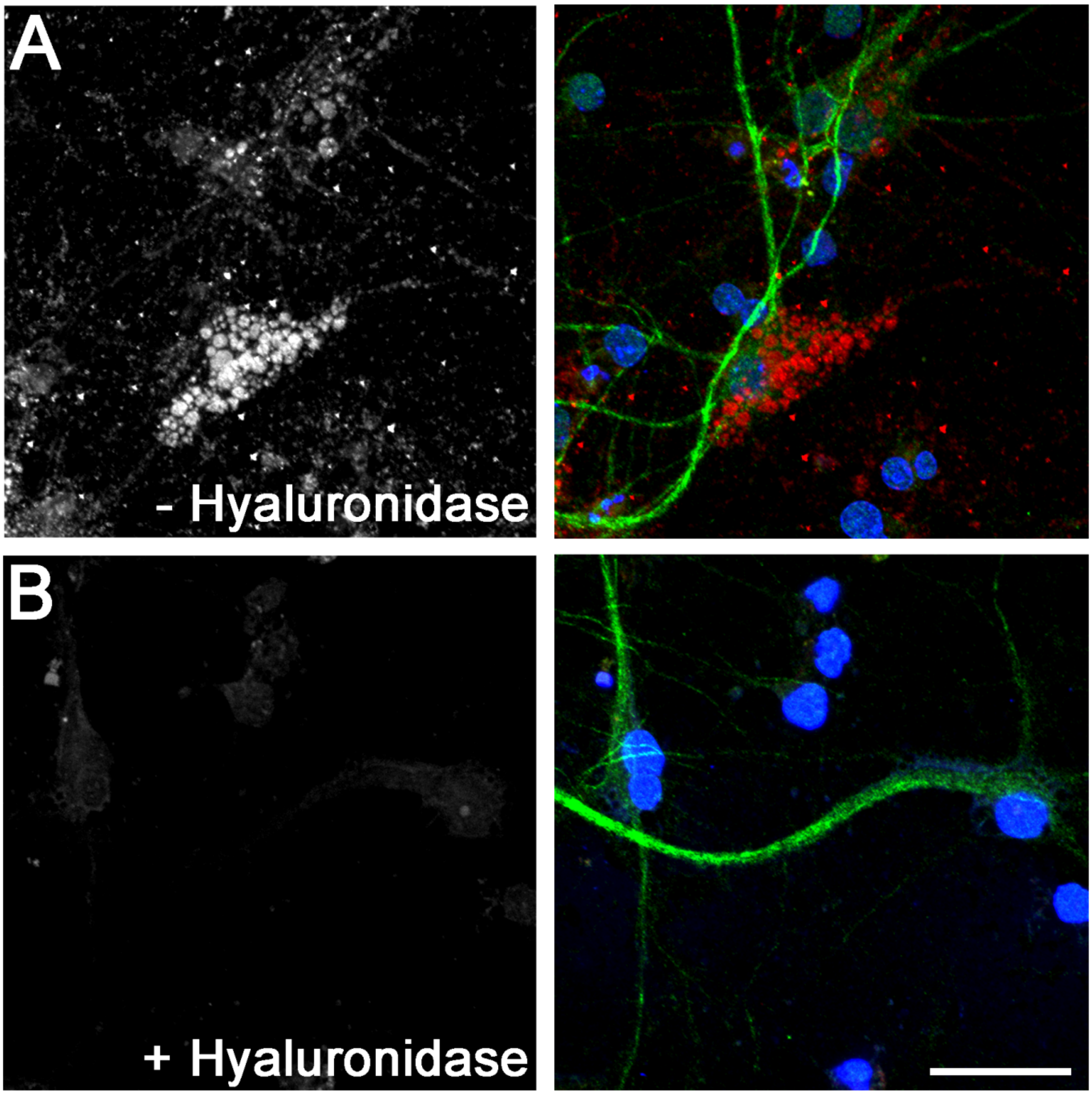

Supplementary Figure S3. Pre-treatment of neurons with hyaluronidase abolishes hyaluronan labelling. Representative examples from neurons at DIV14 are shown. Neurons were stained with bHABP (left: grey, right: red), MAP2 (green), and Hoechst 33258 (blue). (A) HABP labelling with no hyaluronidase pre-treatment. (B) HABP labelling after hyaluronidase pre-treatment. Note that HABP labelling was absent after hyaluronidase pre-treatment. Scale bar: 30 μm.
